# Association of Social Risk Factors With Mortality Among US Adults With a New Cancer Diagnosis

**DOI:** 10.1001/jamanetworkopen.2022.33009

**Published:** 2022-09-16

**Authors:** Matthew P. Banegas, John F. Dickerson, Zhiyuan Zheng, Caitlin C. Murphy, Reginald Tucker-Seeley, James D. Murphy, K. Robin Yabroff

**Affiliations:** 1 Department of Radiation Medicine and Applied Sciences, University of California San Diego, San Diego; 2 Kaiser Permanente Northwest Center for Health Research, Portland, Oregon; 3 Surveillance and Health Equity Science Department, American Cancer Society, Atlanta, Georgia; 4 University of Texas Health Science Center at Houston, School of Public Health, Houston; 5 ZERO—The End of Prostate Cancer, Alexandria, Virginia

## Abstract

This cohort study examines the associations of multiple social risk factors with mortality risk among patients newly diagnosed with cancer in the US.

## Introduction

Social risks, including housing instability and food insecurity, are adverse conditions that may be barriers to health care and can lead to poor health outcomes. Limited research has shown increased mortality risk among cancer survivors with financial hardship.^1,2^ However, less is known about associations between social risks at the time of cancer diagnosis and mortality. We examined the associations of multiple social risks at cancer diagnosis with mortality risk among newly diagnosed adult patients in the US.

## Methods

This cohort study included data from patients 18 years or older who were enrolled at Kaiser Permanente Northwest, were diagnosed with cancer between June 1, 2017, and December 31, 2019, and completed Your Current Life Situation, a social risk survey,^3^ within 90 days before or after diagnosis. The study was approved by the Kaiser Permanente Northwest Center for Health Research Institutional Review Board and was deemed exempt under category 4. The study followed the STROBE reporting guideline.

Patient responses to Your Current Life Situation yielded 4 social risk categories: financial hardship, food insecurity, housing instability, and transportation difficulties (eTable in the Supplement). Race and ethnicity data were collected to account for social and economic barriers that may be imposed by systemic racism. Time to death from any cause was the date of diagnosis to the end of the study period (February 29, 2020). Data were analyzed from October 11, 2021, to January 28, 2022.

Cox proportional hazards regression models were used to measure associations of baseline social risks and mortality for each social risk as well as a combined model including all 4 social risk categories. Propensity score overlap weighting was used for adjusted analyses, whereby logistic regression models estimated the propensity to have each social risk as a function of demographic, socioeconomic, and clinical characteristics (eMethods in the Supplement).^4^ Propensity scores were calculated for each social risk as well as any social risk in the combined model. The proportional hazards assumption was verified with Schoenfeld residuals. Statistical analyses were performed using Stata, version 16.1 (StataCorp LLC).

## Results

The study cohort comprised 1277 patients with a mean (SD) age of 63.5 (13.7); 683 (53.5%) were women. In terms of race and ethnicity, 42 patients (3.3%) were African American, 11 (0.9%) were American Indian or Alaska Native, 65 (5.1%) were Asian American, 63 (4.9%) were Hispanic, 16 (1.2%) were Native Hawaiian or other Pacific Islander, 1074 (84.1%) were non-Hispanic White, and 7 (0.5%) were of other or unspecified race or ethnicity. A total of 1070 patients (83.8%) reported no social risks; 207 (16.2%) reported 1 or more social risks, with financial hardship as the most common (11.5%) (Table 1). A greater proportion of patients with 1 or more social risks were women, were from a racial or ethnic minority group, had Medicaid, had breast or lung cancer, and had advanced-stage tumors (Table 1). There were 275 deaths during the study period. Housing instability was associated with increased mortality risk, both independently (adjusted hazard ratio, 1.54 [95% CI, 1.02-2.31]; *P* = .04) (Table 2) and in the combined model, after adjusting for other social risks (adjusted hazard ratio, 2.05 [95% CI, 1.29-3.27]; *P* = .002).

**Table 1.** Patient Characteristics by Self-Reported Social Risk Status at Baseline^a^

Characteristic	Social risk status	
	≥1 Social risks (n = 207)^b^	No social risks (n = 1070)
Age at diagnosis, y		
18-34	10 (4.8)	27 (2.5)
35-49	33 (15.9)	140 (13.1)
50-64	85 (41.1)	318 (29.7)
65-74	40 (19.3)	349 (32.6)
75-84	31 (15.0)	191 (17.9)
≥85	8 (3.9)	45 (4.2)
Sex		
Female	123 (59.4)	560 (52.3)
Male	84 (40.6)	510 (47.7)
Race and ethnicity^c^		
African American	11 (5.3)	31 (2.9)
American Indian or Alaska Native	2 (1.0)	9 (0.8)
Asian American	17 (8.2)	48 (4.5)
Hispanic	17 (8.2)	46 (4.3)
Native Hawaiian or other Pacific Islander	7 (3.4)	9 (0.8)
Non-Hispanic White	154 (73.9)	920 (86.0)
Other/unspecified	0	7 (0.7)
Household income, median (SD), US$^d^	53 003 (21 506)	61 880 (22 250)
High school education or higher, mean (SD), %^d^	38.9 (14.5)	33.9 (14.7)
Neighborhood Deprivation Index, mean (SD)^e^	0.06 (0.78)	−0.20 (0.65)
Type of first-line treatment^c^		
Systemic therapy^f^	38 (18.4)	183 (17.1)
Surgery	103 (49.8)	625 (58.4)
Radiation	14 (6.8)	44 (4.1)
No treatment	56 (27.1)	237 (22.1)
Cancer site^c^^,^^g^		
Breast	24 (11.6)	94 (8.8)
Colon and/or rectal	23 (11.1)	144 (13.4)
Lung	34 (16.4)	124 (11.6)
Prostate	9 (4.3)	78 (7.3)
Skin	7 (3.4)	20 (1.9)
Other^h^	111 (53.1)	610 (57.0)
Tumor stage		
1	58 (28.0)	283 (26.4)
2	16 (7.7)	138 (12.9)
3	30 (14.5)	127 (11.9)
4	40 (19.3)	165 (15.4)
Unspecified/unstaged^i^	63 (30.4)	357 (33.4)
Follow-up time, median (IQR), mo	10 (4-16)	9 (5-13)
Insurance type		
Commercial	80 (38.6)	390 (36.4)
Medicare	81 (39.1)	626 (58.5)
Medicaid	34 (16.4)	45 (4.2)
Uninsured	12 (5.8)	9 (0.8)
Social risk category^c^		
Financial hardship	147 (11.5)	NA
Food insecurity	81 (6.3)	NA
Housing instability	54 (4.2)	NA
Transportation difficulties	91 (7.1)	NA
Time between YCLS survey and index cancer diagnosis, median (IQR), d	28 (6-50)	23 (1-45)

Abbreviations: NA, not applicable; YCLS, Your Current Life Situation.

^a^ Data are presented as No. (%) of patients unless indicated otherwise. Percentages have been rounded and therefore may not sum to 100.

^b^ Includes patients who reported the presence of either financial hardship, food insecurity, housing instability or transportation difficulties on the YCLS survey at baseline (eMethods in the Supplement).

^c^ Not mutually exclusive; therefore, numbers may sum to more than the subgroup total.

^d^ Based on US Census data.

^e^ The Neighborhood Deprivation Index is a measure of neighborhood socioeconomic context based on 13 US Census–based variables covering domains including poverty, occupation, housing, employment, and education; a higher score indicates higher neighborhood deprivation.

^f^ Includes chemotherapy, hormonal therapy, immunotherapy, and targeted therapy.

^g^ For patients with multiple cancer diagnoses during the study period, we used the first primary diagnosis as the index cancer (1 patient had multiple primary diagnoses on the same diagnosis date).

^h^ Includes cancers in the following site groups: oral cavity and pharynx, digestive system, liver and intrahepatic bile duct, respiratory system, skin excluding basal and squamous, corpus and uterus, male genital system, urinary system, brain and other nervous system, endocrine system, Hodgkin lymphoma, non-Hodgkin lymphoma, lymphocytic leukemia, aleukemic and subleukemic, and miscellaneous, as defined by the National Cancer Institute Surveillance, Epidemiology and End Results Program.

^i^ Includes patients not assigned a tumor stage at diagnosis or for whom stage information at time of diagnosis was unavailable.

**Table 2.** Association of Baseline Social Risks With All-Cause Mortality

Social risk	No. of patients/No. of deaths	Unadjusted estimates		Adjusted estimates^a^	
		Hazard ratio (95% CI)	*P* value	Hazard ratio^2^ (95% CI)	*P* value
Individual risk					
Financial hardship^b^	1217/250	1.04 (0.73-1.49)	.82	0.88 (0.59-1.31)	.53
Food insecurity^b^	1151/234	0.97 (0.60-1.57)	.91	1.02 (0.59-1.75)	.95
Transportation difficulties^b^	1124/232	1.36 (0.81-2.27)	.24	0.96 (0.56-1.65)	.89
Housing instability^b^	1161/248	1.74 (1.20-2.51)	.003	1.54 (1.02-2.31)	.04
Combined model^c^	1277/275	NA	NA	NA	NA
Financial hardship	NA	0.76 (0.46-1.26)	.29	0.64 (0.39-1.05)	.08
Food insecurity	NA	0.70 (0.39-1.29)	.26	0.72 (0.39-1.32)	.29
Transportation difficulties	NA	1.17 (0.67-2.05)	.58	1.11 (0.64-1.92)	.71
Housing instability	NA	2.23 (1.38-3.60)	.001	2.05 (1.29-3.27)	.002

Abbreviations: NA, not applicable; YCLS, Your Current Life Situation.

^a^ Includes propensity score overlap weighting, in which the propensity to have each respective social risk (or any social risk for the combined model) was modeled as a function of age at diagnosis, sex, race and ethnicity, Elixhauser Comorbidity Index, educational attainment, median household income, Neighborhood Deprivation Index, type of first-line cancer treatment, cancer type, tumor stage at diagnosis, insurance type, and days between YCLS survey and incident cancer diagnosis.

^b^ Separate models were run for each of the 4 social risk categories.

^c^ The combined model includes all 4 individual baseline social risk categories. We assessed the presence of multicollinearity between social risk factors using variance inflation factor (VIF), which was well below an established threshold of 10 (mean VIF, 1.42 [range, 1.11-1.63]). Estimates were based on Cox proportional hazards regression. Deaths (n = 275) were observed from date of cancer diagnosis until the end of the study follow-up (February 29, 2020). The presence of a social risk at baseline was defined by patient responses to items on the YCLS (eMethods in the Supplement). For all models, patients with no baseline social risks served as the reference group. Differences in sample sizes between models is due to excluding patients who reported social risks other than the social risk of focus in each respective model. For example, in the model for financial hardship, patients who reported no financial hardship but reported any of the other 3 social risks (food insecurity, transportation difficulties, and/or housing instability) were excluded.

## Discussion

The findings of this cohort study suggest that housing instability was associated with increased mortality risk among US adults with newly diagnosed cancer. Despite national efforts to integrate social risk screening and referral programs within the oncology setting to advance cancer health equity, many community oncology practices have limited social risk screening and referral programs.^5,6^

Study limitations include data from a single health system, limited racial and ethnic diversity among patients, and self-reporting measures that may be subject to recall bias. Regardless, our findings underscore the importance of screening for social risks at the time of cancer diagnosis and connecting patients with relevant social services. Future studies should investigate the effects of social risks on cancer care and subsequent health outcomes to help develop social care strategies and interventions.

## Notes

Supplement. **eTable 1.** Your Current Life Situation (YCLS) Items and Study Social Risk Definitions
eMethods.
