# Supplementary material for: Association of Social Risk Factors With Mortality Among US Adults With a New Cancer Diagnosis
Source: JAMA Netw Open. 2022 Sep 16;5(9):e2233009. doi: 10.1001/jamanetworkopen.2022.33009 (PMC9482059; doi:10.1001/jamanetworkopen.2022.33009)
Supplement: Supplement. — eTable 1. Your Current Life Situation (YCLS) Items and Study Social Risk Definitions eMethods. [file jamanetwopen-e2233009-s001.pdf]

Supplemental Online Content

Banegas MP, Dickerson JF, Zheng Z, et al. Association of social risk factors with mortality among US adults with a new cancer diagnosis. *JAMA Netw Open*. 2022;5(9):e2233009. doi:10.1001/jamanetworkopen.2022.33009

eTable. Your Current Life Situation (YCLS) Items and Study Social Risk Definitions
eMethods.

This supplemental material has been provided by the authors to give readers additional information about their work.

© 2022 Banegas MP et al. *JAMA Network Open.*

eTable. Your Current Life Situation (YCLS) Items and Study Social Risk Definitions

Social Risk	YCLS item	YCLS Responses	Study Social Risk Definition
Financial Hardship	In the past 3 months, did you have trouble paying for any of the following?	Food; Housing; Heat and electricity; Medical needs; Transportation; Childcare; Debts; Other; None of these	Financial Hardship = Yes, if patient selected: Food; Housing OR Heat and electricity OR Medical needs OR Transportation OR Childcare OR Debts OR Other
Food Insecurity	In the past 3 months, how often have you worried that your food would run out before you had money to buy more?	Never; Sometimes; Often; Very often	Food Insecurity = Yes, if patient selected: Sometimes; Often; Very often
Housing Instability	Which of the following best describes your current living situation?	Live alone in my own home (house, apartment, condo, trailer, etc.); may have a pet; Live in a household with other people; Live in a residential facility where meals and household help are routinely provided by paid staff (or could be if requested); Live in a facility such as a nursing home which provides meals and 24-hour nursing care; Temporarily staying with a relative or friend; Temporarily staying in a shelter or homeless; Other	Housing Instability = Yes, if patient selected: Temporarily staying in a shelter or homeless; Temporarily staying with a relative or friend
	Do you have any concerns about your current living situation, like housing conditions, safety, and costs?	Yes; No	Housing Instability = Yes, if patient selected: Yes
Transportation Difficulties	Has lack of transportation kept you from medical appointments or from doing things needed for daily living?	Kept me from medical appointments or from getting medications; Kept me from doing things needed for daily living; Not a problem for me	Transportation Difficulties = Yes, if patient selected: Kept me from medical appointments or from getting medications; Kept me from doing things needed for daily living

© 2022 Banegas MP et al. *JAMA Network Open.*

eMethods

For all Cox regression models, patients with no baseline social risks served as the referent group. This enabled a consistent comparator group across all models. For the combined model, which included all four social risks, we assessed for the presence of multicollinearity between social risk factors using variance inflation factor (VIF), which were well below an established threshold of 10 (mean VIF=1.42, range 1.11-1.63). Adjusted models included propensity-score overlap weighting,[5] in which the propensity to have each respective social risk (or any social risk for the combined model) was modelled as a function of age at diagnosis, gender, race/ethnicity, Elixhauser Comorbidity Index, high school education, median household income, neighborhood deprivation index, type of first-line cancer treatment, cancer type, tumor stage at diagnosis, insurance type, and days between YCLS survey and incident cancer diagnosis. Self-reported race and ethnicity were included in the analysis to account for the differential impact of systemic racism that may impose and sustain social and economic barriers, including social risks, to optimal health outcomes among individuals of different races and ethnicities. Multivariable logistic regression was used for each propensity score model. Death data was obtained from the electronic health record, which is derived from multiple sources including the tumor registry, membership data, state mortality files, and social security administration data. All patients included in the analysis had complete follow-up data. The study was approved by the KPNW Institutional Review Board and was classified as exempt under category 4.

© 2022 Banegas MP et al. *JAMA Network Open.*
